# Supplementary material for: Functional Analysis of Maize SDG102 Gene in Response to Setosphaeria turcica
Source: Plants (Basel). 2025 Nov 13;14(22):3463. doi: 10.3390/plants14223463 (PMC12655767; doi:10.3390/plants14223463)
Supplement: Supplementary file 1 [file plants-14-03463-s001.zip › Supplementary-figures.pdf]

Gene ID	Gene name
Zm00014d035504	ZmPRX2
Zm00014d005104	CER26L
Zm00014d027066	CAD1
Zm00014d030688	ZmPRX44
Zm00014d022332	ZmPRX42
Zm00014d007023	ZmPRX70
Zm00014d021153	ZmPRX44
Zm00014d025050	ZmPRX5
Zm00014d011518	AOGT
Zm00014d033739	ZmPRX65

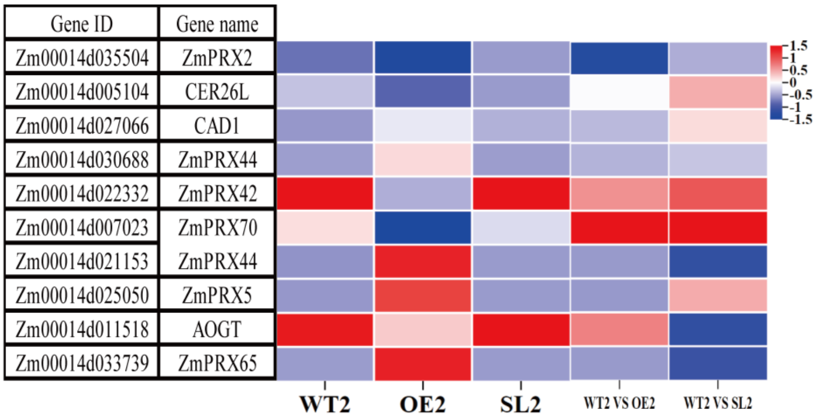

Figure S1 Expression analysis of differentially expressed genes related to the ‘reactive oxygen species generation pathway’.

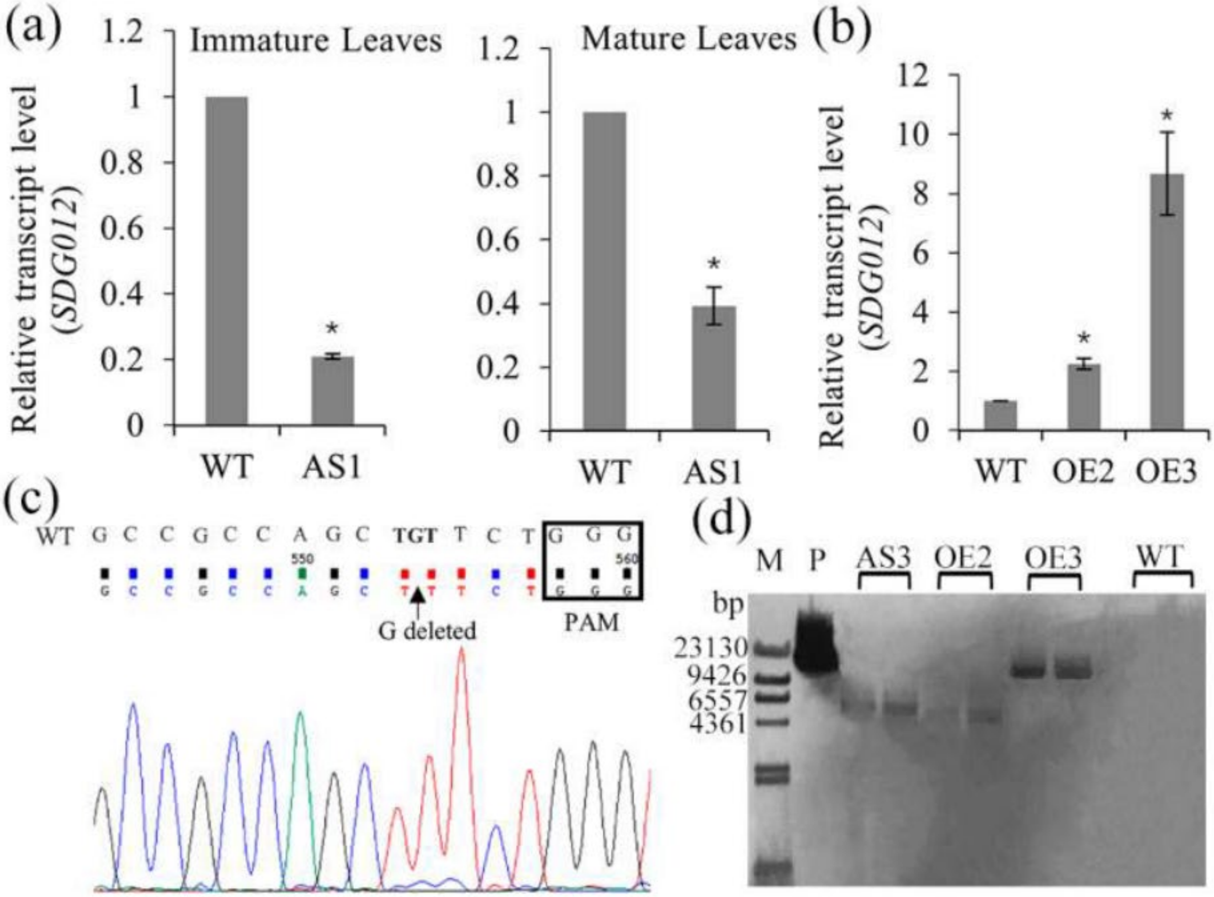

Figure S2 Transgenic Plant Detection

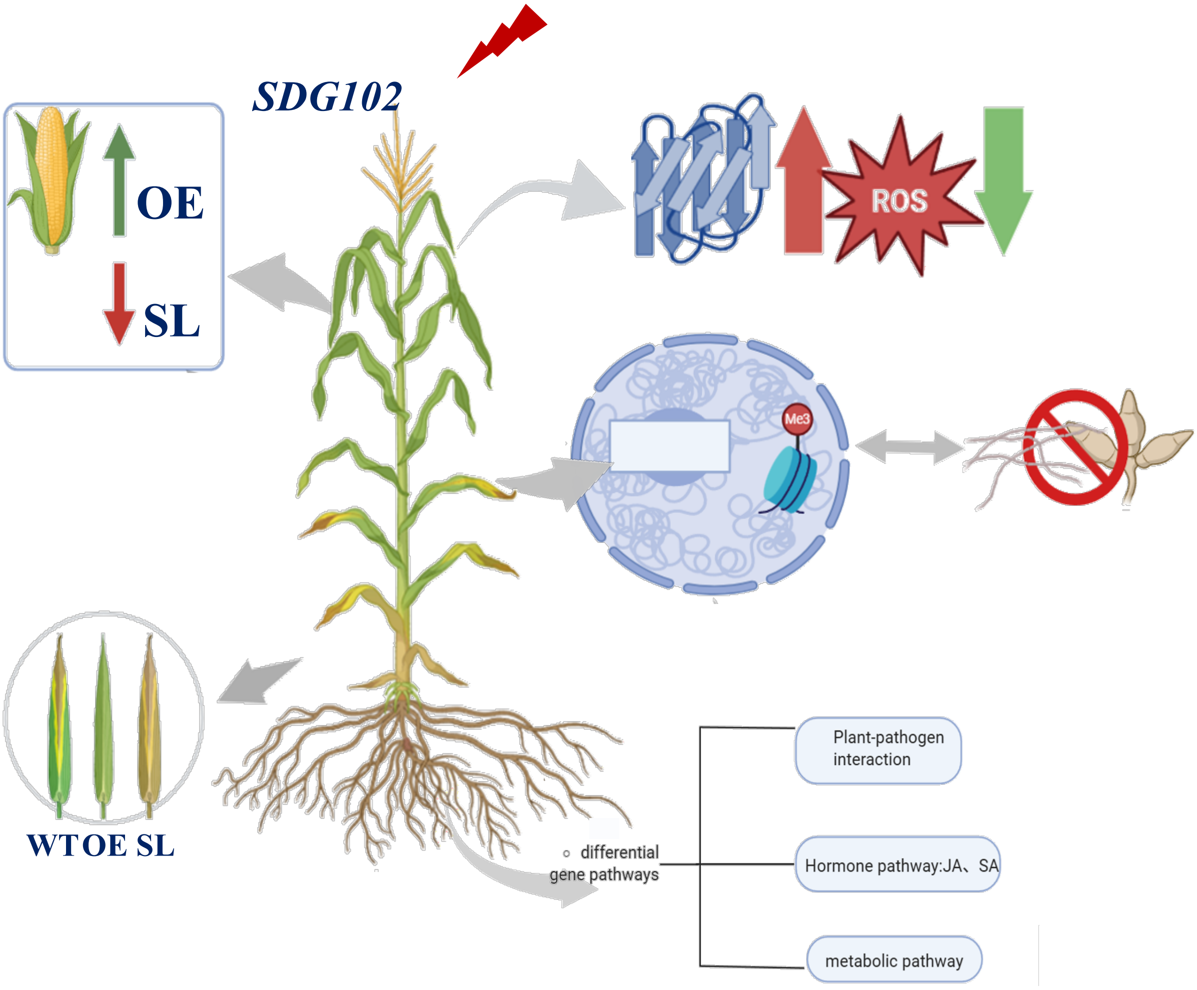

Figure S3 SDG102–mediated defense network
