# Supplementary material for: Functional Analysis of Maize SDG102 Gene in Response to Setosphaeria turcica
Source: Plants (Basel). 2025 Nov 13;14(22):3463. doi: 10.3390/plants14223463 (PMC12655767; doi:10.3390/plants14223463)
Supplement: Supplementary file 1 [file plants-14-03463-s001.zip › Table S1.pdf]

Table S1 Expression levels of differentially regulated genes in WT, OE, and SL genotypes

#ID	Gene name	WT	SL	OE	NR_annotation
Zm00014a013055	SCL9	5.073142333	10.41433133	13.69825233	Scarecrow-like protein 9 [Zea mays]
Zm00014a019507	SCL14	9.738569	21.58830767	19.588057	Scarecrow-like protein 9 [Zea mays]
Zm00014a027310	SCL9	9.290652667	19.52297467	20.01129833	Scarecrow-like protein 9 [Zea mays]
Zm00014a025942	SCL4	5.695129333	1.986121333	1.419135667	Scarecrow-like protein 4 [Zea mays]
Zm00014a034586	IAA4	5.541794333	1.403035333	3.961563667	Auxin-responsive protein IAA4 [Zea mays]
Zm00014a025193	IAA23	19.21799033	6.75019	19.52927967	Auxin-responsive protein IAA23 [Zea mays]
Zm00014a033479	IAA20	7.650276	2.194652	0.074536333	Auxin-responsive protein IAA20 [Zea mays]
Zm00014a033039	SAUR40	2.444338667	42.666199	37.88289633	Auxin-responsive protein SAUR40 [Zea mays]
Zm00014a016766	PRK5	2.226250667	0	0	putative LRR receptor-like serine/threonine-protein kinase [Zea mays]
Zm00014a041816	PRK3	7.570133	0	0.064929333	Pollen receptor-like kinase 3 [Zea mays]
Zm00014a026632	LRR	8.455556	0	0.046736333	probable LRR receptor-like serine/threonine-protein kinase At4g31250 [Zea mays]
Zm00014a021930	ARF14	2.356965667	0.717366667	1.490362667	unknown [Zea mays]
Zm00014a024168	ARF19	0.446068667	1.510761333	2.377939	Auxin response factor 19 [Zea mays]
Zm00014a024479	ARF5	0.493062333	2.334820667	1.865818	Auxin response factor 5 [Zea mays]
Zm00014a003475	ARF2	3.988814	7.586069667	8.923424333	Auxin response factor 2 [Zea mays]
Zm00014a021930	ARF14	2.356965667	0.717366667	1.490362667	unknown [Zea mays]
Zm00014a023233	ARF22	8.704547667	1.891796	7.056628667	Auxin response factor 22 [Zea mays]
Zm00014a006184	IV.1	9.919761333	4.189712	7.627414	L-type lectin-domain containing receptor kinase IV.1 [Zea mays]
Zm00014a014378	Lr10	6.325692667	1.770409333	7.295456667	Rust resistance kinase Lr10 [Zea mays]
Zm00014a038250	MYB EFM	1.445492333	3.637500667	4.802863667	Myb family transcription factor EFM [Zea mays]
Zm00014a002957	MYB EFM	2.715867	7.850347667	11.88248667	hypothetical protein Zm00014a_002957 [Zea mays]
Zm00014a043351	MYB	5.851518667	2.367402333	5.192075	putative Myb family transcription factor [Zea mays]

Zm00014a033636	DOGL3	0.277513	2.325821333	0.781653	Protein DOG1-like 3 [Zea mays]
Zm00014a042862	BHLH41	0.999035667	3.65444	3.039458333	putative transcription factor bHLH041 [Zea mays]
Zm00014a015136	bHLH66	6.625395667	24.57049733	3.913947	Transcription factor bHLH66 [Zea mays]
Zm00014a039584	bHLH3	0.041046667	1.077499	2.239529333	Transcription factor bHLH3 [Zea mays]
Zm00014a017907	TIFY 11b	29.294972	0	0	Protein TIFY 11b [Zea mays]
Zm00014a026934	GH3.2	3.420472333	0	0	putative indole-3-acetic acid-amido synthetase GH3.2 [Zea mays]
Zm00014a001879	CXE15	3.323704333	0.349769	2.871901667	putative carboxylesterase 15 [Zea mays]
Zm00014a003101	CXE15	23.00433433	9.313818667	24.37994067	putative carboxylesterase 15 [Zea mays]
Zm00014a041465		0.039653	2.586458	1.852482333	hypothetical protein Zm00014a_041465 [Zea mays]
Zm00014a034136	CPRF2	19.012426	50.350637	12.161379	Light-inducible protein CPRF2 [Zea mays]
Zm00014a042290		29.066545	61.81580133	89.87119267	SAUR12 - auxin-responsive SAUR family member [Zea mays]
Zm00014a029234	DWARF8	7.697473333	3.414050333	6.692611333	DELLA protein DWARF8 [Zea mays]
Zm00014a010963	LOC100217048	0	4.588019	1.827992	uncharacterized protein LOC100217048 [Zea mays]
Zm00014a040840	LOC100284894	0.468467333	8.316392667	9.300734333	uncharacterized protein LOC100284894 [Zea mays]
Zm00014a035672	F-box1	2.987295333	5.756579	9.145642333	EIN3-binding F-box protein 1 [Zea mays]
Zm00014a039091	LG2	2.517400333	4.948715	8.676241667	Transcription factor LG2 [Zea mays]
Zm00014a000681	CXE18	1.344654333	5.039573667	44.200591	putative carboxylesterase 18 [Zea mays]
Zm00014a041511	PYL4	23.586689	56.206131	30.029604	Abscisic acid receptor PYL4 [Zea mays]
Zm00014a000120		45.25142133	0.058344	1.331091667	Pathogenesis-related protein 1A [Zea mays]
Zm00014a010337	TIFY 11b	12.569671	67.92824067	77.86365533	Protein TIFY 11b [Zea mays]
Zm00014a023069	TIFY10C	20.306484	7.649140667	1.352364333	hypothetical protein Zm00014a_023069 [Zea mays]
Zm00014a039252	PIL13	2.068875667	4.596165667	13.673132	hypothetical protein Zm00014a_039252 [Zea mays]
Zm00014a023027	ERF1B	4.255097333	14.66126767	16.86359733	Ethylene-responsive transcription factor 1B [Zea mays]
Zm00014a021260	TGAL6	12.34215833	2.464499333	27.432105	hypothetical protein Zm00014a_021260 [Zea mays]
Zm00014a030760	TGAL7	4.591619667	0.840667	1.342148333	Transcription factor TGAL7 [Zea mays]
Zm00014a030503		38.132696	0.022473333	0	Pathogenesis-related protein 1 [Zea mays]

Zm00014a044689	CTR1	4.299423333	1.245857	5.750453333	Serine/threonine-protein kinase CTR1 [Zea mays]
Zm00014a022194		2.734119	6.819205	14.693223	hypothetical protein Zm00014a_022194 [Zea mays]
Zm00014a029183	MAPK12	0.333221	1.284136	0.669539	Mitogen-activated protein kinase kinase kinase 12 [Zea mays]
Zm00014a029003	IV.1	2.907346667	0.522076333	0.597514	L-type lectin-domain containing receptor kinase IV.1 [Zea mays]
Zm00014a004001	PP2C06	7.026002333	2.550622667	4.602245333	putative protein phosphatase 2C 6 [Zea mays]
Zm00014a006815	SD2-5	5.600856333	1.852561667	5.357144667	G-type lectin S-receptor-like serine/threonine-protein kinase SD2-5 [Zea mays]
Zm00014a006816	SD2-5	3.088565333	10.365257	17.78273733	G-type lectin S-receptor-like serine/threonine-protein kinase SD2-5 [Zea mays]
Zm00014a023703	SD2-5	14.48421867	6.001109667	23.16389367	G-type lectin S-receptor-like serine/threonine-protein kinase SD2-5 [Zea mays]
