# Supplementary material for: Functional Analysis of Maize SDG102 Gene in Response to Setosphaeria turcica
Source: Plants (Basel). 2025 Nov 13;14(22):3463. doi: 10.3390/plants14223463 (PMC12655767; doi:10.3390/plants14223463)
Supplement: Supplementary file 1 [file plants-14-03463-s001.zip › Table S2.pdf]

Table S2 Primers for 12 pathogen immunity-related defense genes screened from the target module

Gene ID	F	R
Zm00014d028728	AGCCATCGCTTCCGTAAGTT	ACAACTGAACACAGCGCGAG
Zm00014d044630	CGTACGCTCTGCACGAGAA	GCTTCTTGGCGTCCTTCTTC
Zm00014d015487	GCAAGGCACATACCCTGTCT	ACTATTCCTTGCAGGCCACC
Zm00014d008670	CCTGGAGATCCCCACTGAGA	CTTGTCCGTCCAGGTGTTGA
Zm00014d009572	CTGGCGTAGCCAGGAAGATAG	GCTGGGTTTCTCCTCGATCC
Zm00014d000681	GGCAGTGTCTTCTGGTGACT	TTGGTGTCGATGGGCTTGAA
Zm00014d043919	CTTCGCCATCATCGGCAAC	GCATGTATCTTACGCCTGGCT
Zm00014d031552	TGCATATCGACAGGACCACA	TGGGCCAGTCAAAGGAATCT
Zm00014d027196	ACCTACGTCGCCAACTACAA	AAATCCTCATCGATGGCCCA
Zm00014d023813	AACATCTCTATCGCGGCTGA	CATTCATGCCACCTGATCCG
Zm00014d009653	GCGAAAATGATGGCCTCCTT	CTTGCTTTCCGGCTTGAAGT
Zm00014d018747	ACCTCTCCGACATCTGCATC	TCCTTGGTGAGCATGGACAT
